# Survival Outcomes and Prognostic Factors of Central Nervous System Malignancies in Adult Patients: A Single-Center Retrospective Cohort Study From a Tertiary Hospital in Bangladesh

**DOI:** 10.7759/cureus.112856

**Published:** 2026-07-17

**Authors:** Md. Arifur Rahman, Patoary M Faruque, Md. Raziul Haque, A S M Fazlul Karim Chowdhury, Numair Elahi, Qamruzzaman Chowdhury

**Affiliations:** 1 Department of Oncology and Radiotherapy, Bangladesh Specialized Hospital, Dhaka, BGD; 2 Department of Neurosurgery, Bangladesh Specialized Hospital, Dhaka, BGD; 3 Department of Bioinformatics, Heal Holmes Ltd, Dhaka, BGD; 4 Department of Chemistry and Chemical Biology, Northeastern University, Boston, USA

**Keywords:** bangladesh, central nervous system malignancies, glioblastoma, kaplan–meier analysis, low- and middle-income countries, overall survival, prognostic factors, radiotherapy

## Abstract

Background and objective

Central nervous system (CNS) malignancies carry a high mortality burden, and longitudinal survival data from low- and middle-income countries remain sparse. We aimed to characterise overall survival (OS) and identify key prognostic factors in adult patients with primary CNS malignancies managed at a tertiary referral hospital in Bangladesh.

Methods

This retrospective cohort study included consecutive adult patients aged 18 years or older with histopathologically confirmed primary CNS malignancies treated between June 2018 and December 2023. Patients with severe, life-limiting comorbidities, as defined in the Methods section, and those lost to follow-up within 30 days were excluded. OS was estimated using the Kaplan-Meier method, group differences were assessed by the log-rank test, and a multivariable Cox proportional hazards model was used to identify independent prognostic factors. Reporting followed the Strengthening the Reporting of Observational Studies in Epidemiology (STROBE) guidelines.

Results

A total of 151 adult patients were analysed (mean age: 47.3 ± 13.8 years; 100 (66.2%) men, 51 (33.8%) women; male-to-female ratio: 1.96:1). Median follow-up among censored patients was 29.3 months (interquartile range: 10.4-43.7), and 47 (31.1%) patients remained alive at last follow-up. Median OS for the entire cohort was 19.6 months (95% confidence interval (CI): 14.3-27.6). Histopathological diagnosis stratified survival (log-rank p=0.003): median OS was 12.4 months (95% CI: 9.2-19.6) for glioblastoma (n=79), 21.9 months (95% CI: 14.7-39.7) for grade II-III astrocytoma (n=51), 113.8 months (95% CI: 1.1-113.8) for ependymoma (n=7), and 153.3 months (95% CI: 24.4-153.3) for oligodendroglioma (n=14); the latter two estimates are based on small event numbers and should be interpreted with caution. World Health Organization (WHO) tumour grade stratified survival (log-rank p=0.003): median OS was 55.0 months (95% CI: 27.8-124.8) for grade II (n=41), 24.4 months (95% CI: 19.2-66.7) for grade III (n=22), and 12.2 months (95% CI: 9.2-17.9) for grade IV (n=88). Age was the strongest determinant (log-rank p<0.001): median OS was 66.7 months (95% CI: 38.5-124.8) for patients aged 18-40 years (n=48), 18.1 months (95% CI: 12.1-24.4) for those aged 41-60 years (n=73), and 7.7 months (95% CI: 5.4-9.8) for those aged 60 years or older (n=30). The extent of surgical resection was not associated with survival (log-rank p=0.620). On multivariable Cox regression, each 10-year increment in age conferred an adjusted hazard ratio of 1.84 (95% CI: 1.52-2.23, p<0.001), the only independent predictor of mortality.

Conclusion

Survival from adult CNS malignancies in this Bangladeshi cohort remained lower than benchmarks reported from high-income settings, particularly for glioblastoma. Age was the dominant independent prognostic factor. The findings underscore the urgent need for routine molecular diagnostics, expanded radiotherapy and adjuvant chemotherapy capacity, and a national cancer registry to guide neuro-oncological care in Bangladesh.

## Introduction

Central nervous system (CNS) malignancies are a heterogeneous group of neoplasms affecting the brain and spinal cord. Although they account for only a small fraction of cancers diagnosed annually worldwide, their impact on public health is disproportionately severe owing to the high mortality of high-grade subtypes and the profound morbidity associated with their anatomical location in eloquent neural structures [1]. The global age-adjusted incidence of primary malignant brain tumours has been estimated at approximately 3.5 per 100,000 population, with marked geographical variation [2].

A substantial disparity exists between the management of CNS tumours in high-income countries (HICs) and in low- and middle-income countries (LMICs). In HICs, neuro-oncology has shifted from purely anatomical and histological classification to integrated molecular diagnosis. The 2021 World Health Organization (WHO) Classification of Tumours of the CNS mandates evaluation of isocitrate dehydrogenase (IDH) mutation status, 1p/19q codeletion, and O6-methylguanine-DNA methyltransferase (MGMT) promoter methylation to define entities such as glioblastoma and oligodendroglioma [3]. This molecular stratification has enabled personalised therapy and incremental improvements in survival.

In contrast, in LMICs such as Bangladesh, the infrastructure required to support modern neuro-oncological care, including neuro-navigation, intensity-modulated radiation therapy, and molecular pathology, remains accessible to only a fraction of the population [4]. As a result, survival statistics from this region largely reflect the natural history of disease modified by basic surgical and radiotherapeutic interventions rather than the molecularly driven outcomes that define contemporary Western trials.

Bangladesh does not yet have a comprehensive population-based national cancer registry [5]. The available epidemiological data on CNS malignancies are largely derived from fragmented hospital-based registries. Earlier work from major institutions has identified astrocytomas and meningiomas as the predominant histological subtypes and reported a male preponderance [6,7]. However, these studies have focused predominantly on incidence patterns and immediate surgical outcomes, with a critical paucity of longitudinal survival data.

This study aimed to address that knowledge gap by providing a robust survival analysis for adult patients with primary CNS malignancies at a single tertiary referral hospital in Bangladesh. Specifically, we sought to (1) estimate the median overall survival (OS) for adult patients with CNS malignancies in this cohort; (2) evaluate the impact of age, sex, histological subtype, and tumour grade on survival; (3) quantify the survival contribution of radiotherapy and varying degrees of surgical resection; and (4) contextualise these findings against global and regional data. The term "glioblastoma" is used throughout for WHO grade IV astrocytic tumours, replacing the outdated descriptor "glioblastoma multiforme", in keeping with the WHO 2021 classification [3].

## Materials and methods

Study design and setting

This retrospective single-centre cohort study was conducted at a tertiary care hospital in Bangladesh that serves both urban and rural districts as a referral centre for complex neurosurgical and oncological care. The study period spanned 1 June 2018 to 31 December 2023. Reporting of the study adhered to the Strengthening the Reporting of Observational Studies in Epidemiology (STROBE) guidelines for cohort studies [8].

Ethical approval

The study protocol was reviewed and approved by the Institutional Review Board (IRB) of Bangladesh Specialized Hospital (reference BSHL/Ethical Clearance/2025/11, dated 19 November 2025) and was conducted in accordance with the principles of the Declaration of Helsinki. Given the retrospective nature of the study, the IRB granted a waiver of individual informed consent. Ethics approval was obtained before formal data extraction and analysis. All patient identifiers were removed and replaced with anonymised study codes before analysis.

Study population

Consecutive adult patients aged 18 years or older with a primary diagnosis of CNS malignancy who were managed at the study hospital during the inclusion window were screened. After application of inclusion and exclusion criteria, 151 adult patients were retained for analysis.

Inclusion criteria

Eligible patients were 18 years of age or older, had a histopathologically confirmed primary CNS malignancy, received at least one modality of active oncological treatment (surgery, chemotherapy, or radiotherapy) at the study institution, and had medical records containing demographic, operative, histopathological, and follow-up data sufficient for survival analysis.

Exclusion criteria

Patients were excluded if they (a) were younger than 18 years; (b) had a histologically benign CNS tumour without evidence of malignant behaviour; (c) had incomplete records or were lost to follow-up within 30 days of diagnosis; or (d) had a severe, life-limiting comorbidity as defined below. Three otherwise eligible adult patients with a negative recorded follow-up time due to a data-entry error were further excluded from the time-to-event analysis. In line with established frameworks for oncology trial eligibility [9,10], excluded comorbidities were operationalised through condition-specific staging systems: New York Heart Association (NYHA) class III or IV heart failure or myocardial infarction within the previous six months; severe chronic obstructive pulmonary disease corresponding to Global Initiative for Chronic Obstructive Lung Disease (GOLD) stage 4 or requiring long-term home oxygen therapy; end-stage renal disease on chronic dialysis; decompensated cirrhosis (Child-Pugh class B or C); and active non-CNS malignancy diagnosed within the preceding five years. Each condition was therefore captured using its own staging system rather than a single non-cancer comorbidity classifier.

Data collection and variables

Two trained reviewers extracted data from physical case notes and the electronic medical record system into a structured proforma. Variables included demographic data (age at diagnosis, sex), tumour characteristics (histopathological subtype per WHO classification, grade II to IV, anatomical location, laterality, IDH1/IDH2 status when available), treatment data (type of surgery: gross total resection (GTR), subtotal resection (STR), near-total resection (NTR), or stereotactic/open biopsy; radiotherapy status and prescription dose; adjuvant chemotherapy status), and outcome data. OS was defined as the time, in months, from the date of histopathological diagnosis to death from any cause; patients alive at last follow-up were censored on the date of last contact.

Because routine IDH1/IDH2 mutation testing, 1p/19q codeletion analysis, and MGMT promoter methylation assessment were not universally available at the study institution, all tumour classifications in this study are based on integrated histomorphological criteria per the 2016 WHO framework and should be interpreted as morphological approximations of the 2021 WHO-integrated diagnoses. Tumour grades are reported using Roman numerals (grade II-IV) consistent with predominant usage in the cited literature; where 2021 WHO-compliant entity names are used, Arabic numerals are applied (e.g., glioblastoma, grade IV, and IDH-wildtype).

Age group definitions

Age was analysed both as a continuous variable per 10-year increment and as a categorical variable in three strata: young adults (18-40 years), middle-aged adults (41-60 years), and senior adults (60 years or older), in line with conventional adult neuro-oncology stratification.

Statistical analysis

Continuous variables are summarised as mean ± standard deviation or median with interquartile range (IQR); categorical variables are expressed as frequencies and percentages. Survival probabilities were estimated using the Kaplan-Meier method, and group differences were evaluated by the log-rank test. Multivariable Cox proportional hazards regression was used to estimate adjusted hazard ratios (HRs) with 95% confidence intervals (CI), entering age (per 10-year increment), sex, WHO grade IV status, receipt of radiotherapy, receipt of adjuvant chemotherapy, and gross resection (GTR or STR) versus less-than-gross resection (NTR or biopsy). The proportional hazards assumption was assessed using scaled Schoenfeld residuals; no significant time dependence was detected for any covariate (all individual p>0.18; global test p=0.335), confirming the overall validity of the model. Full Schoenfeld residual results are provided in Appendix A. The median follow-up duration was computed using the reverse Kaplan-Meier approach and is reported with IQR; the censoring rate was reported as the proportion of patients alive at last follow-up. A two-sided p-value of less than 0.05 was considered statistically significant. All analyses were performed using Python (version 3.12; Python Software Foundation, Beaverton, OR, USA) with the lifelines library (version 0.27; open-source, available at: https://lifelines.readthedocs.io) for survival modelling and pandas for data handling. Analytical code is available from the corresponding author on reasonable request.

## Results

Cohort characteristics

The final analytical cohort consisted of 151 adult patients with histopathologically confirmed primary CNS malignancies. The mean age at diagnosis was 47.3 ± 13.8 years (median: 47.7 years; IQR: 37.2-58.2 years; range: 18.0-79.8 years). The cohort included 100 (66.2%) men and 51 (33.8%) women, corresponding to a male-to-female ratio of 1.96:1. By age stratum, 48 (31.8%) patients were young adults aged 18-40 years, 73 (48.3%) were middle-aged adults aged 41-60 years, and 30 (19.9%) were senior adults aged 60 years or older. Glioblastoma was the most frequent histopathological diagnosis, present in 79 (52.3%) patients, followed by grade II-III astrocytoma in 51 (33.8%) patients, oligodendroglioma in 14 (9.3%) patients, and ependymoma in 7 (4.6%) patients. Adjuvant external-beam radiotherapy was administered to 94 (62.3%) patients, and adjuvant systemic chemotherapy was administered to 52 (34.4%) patients. The baseline characteristics of the cohort are summarised in Table 1.

**Table 1:** Baseline demographic, tumour, and treatment characteristics of the adult CNS malignancy cohort (n=151) Stereotactic and open biopsy are combined under "biopsy." CNS, central nervous system; IQR, interquartile range; SD, standard deviation; WHO, World Health Organization.

Characteristic	Value	n	%
Mean age ± SD (years)	47.3 ± 13.8	—	—
Median age, IQR (years)	47.7 (37.2-58.2)	—	—
Age range (years)	18.0-79.8	—	—
Sex			
Male		100	66.2
Female		51	33.8
Age group			
18-40 years		48	31.8
41-60 years		73	48.3
≥60 years		30	19.9
Histopathological diagnosis			
Glioblastoma (WHO grade IV)		79	52.3
Astrocytoma (grade II-III)		51	33.8
Oligodendroglioma		14	9.3
Ependymoma		7	4.6
WHO tumour grade			
Grade II		41	27.2
Grade III		22	14.6
Grade IV		88	58.3
Extent of surgical resection			
Gross total resection		107	70.9
Subtotal resection		23	15.2
Near-total resection		12	7.9
Biopsy		9	6.0
Adjuvant radiotherapy received		94	62.3
Adjuvant chemotherapy received		52	34.4
Status at last follow-up			
Deceased		104	68.9
Alive (censored)		47	31.1

OS

**Figure 1:**
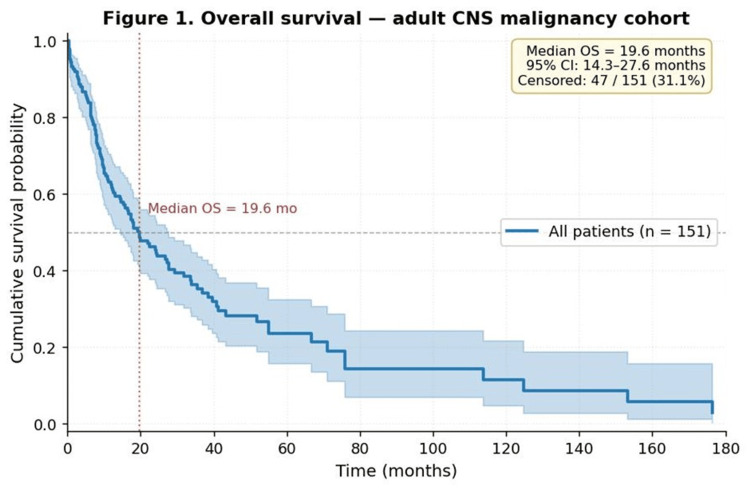
Kaplan-Meier overall survival curve for the adult CNS malignancy cohort (n=151; 104 events) Median OS was 19.6 months (95% CI: 14.3-27.6 months). Forty-seven (31.1%) patients were censored at last follow-up; tick marks on the curve indicate censoring events. The shaded band represents the 95% CI around the survival estimate. CI, confidence interval; CNS, central nervous system; OS, overall survival.

At the end of the study window, 104 of the 151 (68.9%) patients had died, and 47 (31.1%) patients remained alive and were censored at the date of last follow-up. The median follow-up duration among censored patients was 29.3 months (IQR: 10.4-43.7), and the longest observed follow-up was 177 months. The median OS for the entire adult cohort was 19.6 months (95% CI: 14.3-27.6), confirming the marked heterogeneity that characterises CNS malignancy outcomes. The Kaplan-Meier curve for the whole cohort, with the median-survival reference line and tick marks denoting censored observations, is shown in Figure 1.

Survival by age group

**Figure 2:**
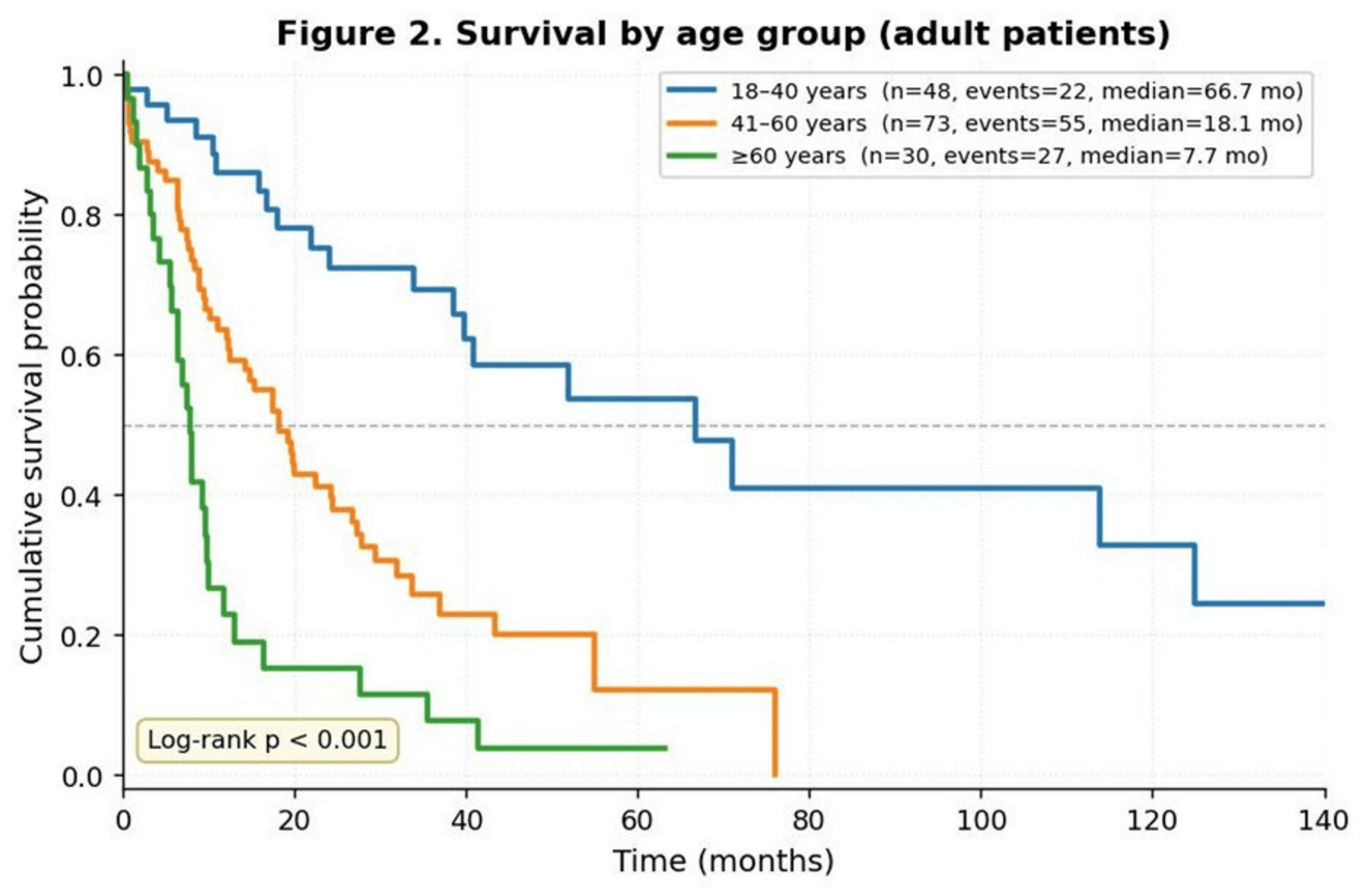
Kaplan-Meier overall survival curves stratified by age group in the adult CNS malignancy cohort Strata: 18-40 years (n=48; 22 events; median: 66.7 months), 41-60 years (n=73; 55 events; median: 18.1 months), and ≥60 years (n=30; 27 events; median: 7.7 months). Overall log-rank p<0.001. The number-at-risk table below the curves shows residual cohort size at 28-month intervals. CNS, central nervous system.

Age was the strongest stratifier of survival. Young adult patients aged 18-40 years (n=48) had a median OS of 66.7 months, approximately 3.7 times longer than middle-aged adults aged 41-60 years (n=73; median: 18.1 months) and 8.6 times longer than senior adults aged 60 years or older (n=30; median: 7.7 months). The difference across the three age strata was highly significant (log-rank p<0.001). The numerical estimates are summarised in Table 2, and the corresponding Kaplan-Meier curves with the residual-at-risk table are presented in Figure 2.

**Table 2:** Median OS of adult patients with CNS malignancies stratified by age group Overall log-rank p<0.001 across the three groups CI, confidence interval; CNS, central nervous system; OS, overall survival.

Age group	n	Events	Median OS (months)	95% CI
Young adults (18-40 years)	48	22	66.7	38.5-124.8
Middle-aged adults (41-60 years)	73	55	18.1	12.1-24.4
Senior adults (≥60 years)	30	27	7.7	5.4-9.8

Survival by histopathological diagnosis

**Figure 3:**
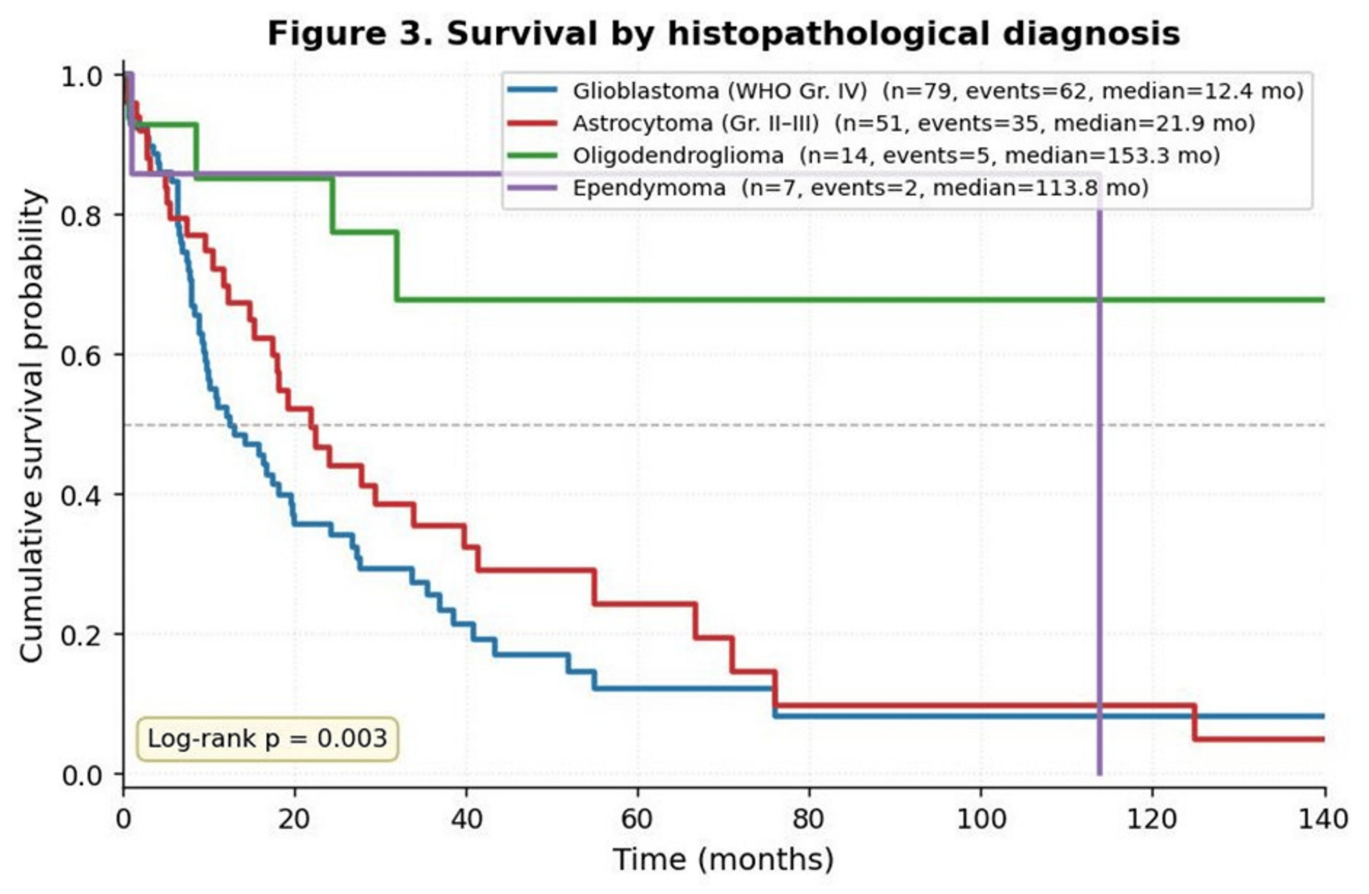
Kaplan-Meier overall survival curves stratified by histopathological diagnosis Strata: glioblastoma, WHO grade IV (n=79; 62 events; median: 12.4 months); astrocytoma, grade II-III (n=51; 35 events; median: 21.9 months); oligodendroglioma (n=14; 5 events; median: 153.3 months); and ependymoma (n=7; 2 events; median: 113.8 months). Log-rank p=0.003 WHO, World Health Organization.

Histopathological diagnosis was a significant predictor of OS (log-rank p=0.003). Patients with oligodendroglioma exhibited the longest median survival (153.3 months), consistent with the favourable biology of these tumours and their established sensitivity to alkylating chemotherapy regimens [11]. Patients with ependymoma also showed durable survival (median: 113.8 months). It should be noted that both the oligodendroglioma and ependymoma median OS estimates are technically valid Kaplan-Meier values but are driven by single long-term events occurring in the last patient remaining at risk within each subgroup (5 events in 14 patients and 2 events in 7 patients, respectively); readers should rely primarily on the 95% CIs rather than the point estimates for these small subgroups. Patients with grade II-III astrocytoma had an intermediate outcome (21.9 months), whereas patients with glioblastoma had the shortest median OS (12.4 months). The numerical estimates are summarised in Table 3, and the corresponding Kaplan-Meier curves are presented in Figure 3.

**Table 3:** Distribution of adult CNS malignancies by histopathological subtype with median OS p-values were calculated using the log-rank test to compare each subtype with the glioblastoma reference group. The overall log-rank test across the four histopathological groups was p=0.003. †Median OS for oligodendroglioma and ependymoma is derived from the final event coinciding with the last patient at risk in each subgroup. These estimates are statistically fragile; the 95% CI should be used for clinical interpretation. CI, confidence interval; CNS, central nervous system; OS, overall survival; Ref, reference category for log-rank comparison; WHO, World Health Organization.

Histopathological subtype	n	% of cohort	Median OS (months)	95% CI	p-value
Glioblastoma (WHO grade IV)	79	52.3	12.4	9.2-19.6	Ref
Astrocytoma (grade II-III)	51	33.8	21.9	14.7-39.7	0.013
Oligodendroglioma^†^	14	9.3	153.3	24.4-153.3	<0.001
Ependymoma^†^	7	4.6	113.8	1.1-113.8	0.072
Total cohort	151	100.0	19.6	14.3-27.6	—

Survival by WHO tumour grade

**Figure 4:**
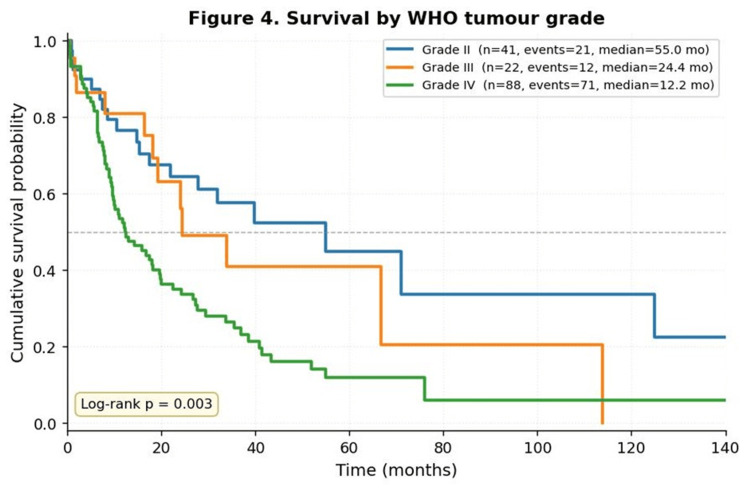
Kaplan-Meier overall survival curves stratified by WHO tumour grade Strata: grade II (n=41; 21 events; median: 55.0 months), grade III (n=22; 12 events; median: 24.4 months), and grade IV (n=88; 71 events; median: 12.2 months). Higher grade was associated with significantly shorter survival (log-rank p=0.003). WHO, World Health Organization.

Tumour grade was a significant determinant of survival (log-rank p=0.003). As anticipated, patients with WHO grade II tumours had the longest median survival (55.0 months; n=41), followed by patients with grade III tumours (24.4 months; n=22) and patients with grade IV tumours (12.2 months; n=88). Because all adult malignant CNS tumours in the cohort fell into grades II to IV after the exclusion of histologically benign lesions, a separate grade I category is not presented; this also resolves the previously reported paradoxical grade I estimate, which had reflected very small subgroup contamination in the pre-filtered dataset. The corresponding Kaplan-Meier curves with the residual-at-risk table are presented in Figure 4.

Survival by extent of surgical resection

**Figure 5:**
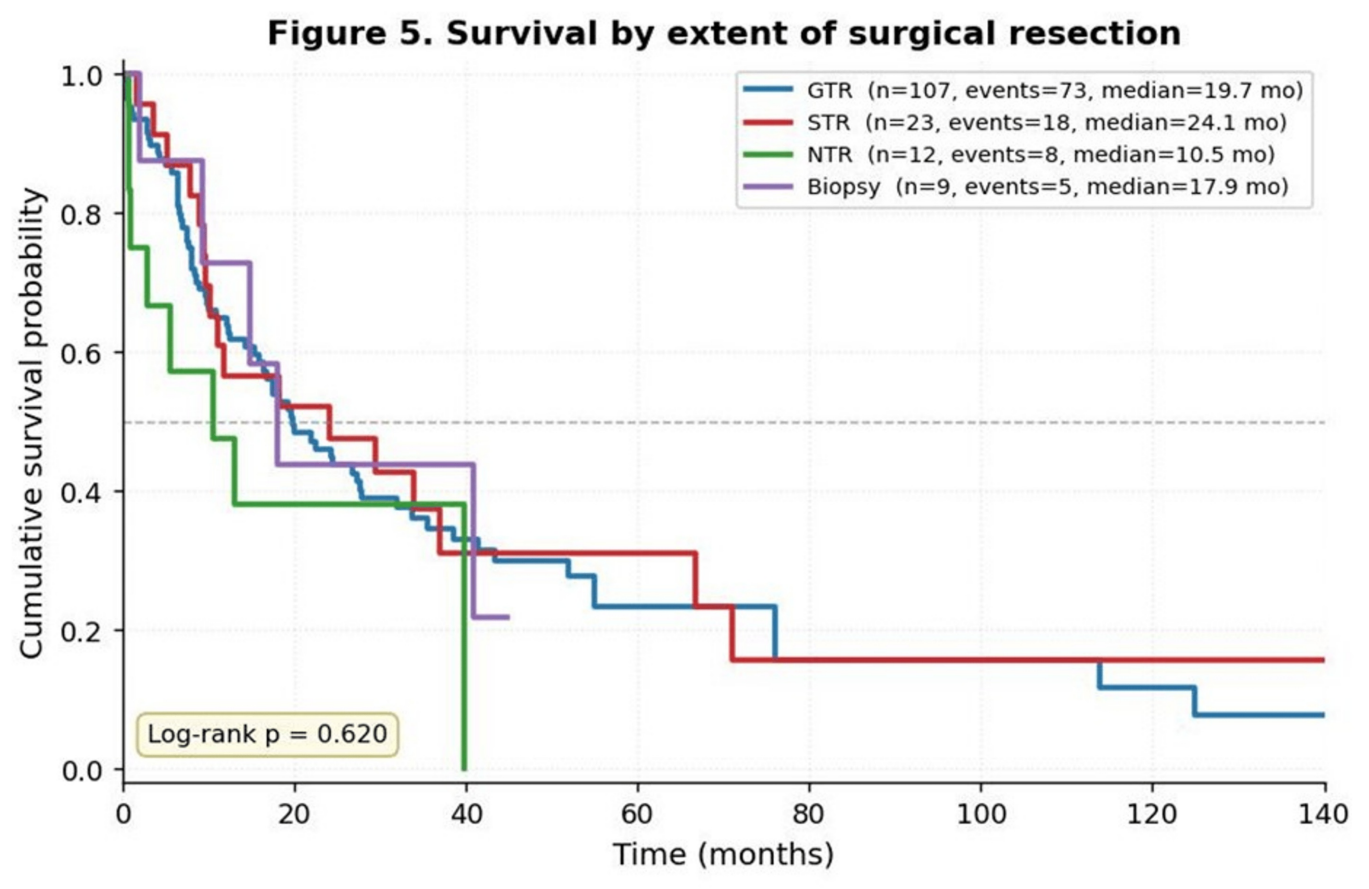
Kaplan-Meier overall survival curves stratified by extent of surgical resection Strata: GTR (n=107; 73 events; median: 19.7 months), STR (n=23; 18 events; median: 24.1 months), NTR (n=12; 8 events; median: 10.5 months), and biopsy (n=9; 5 events; median: 17.9 months). Log-rank p=0.620 GTR, gross total resection; NTR, near-total resection; STR, subtotal resection.

The extent of surgical resection achieved at the primary operation was categorised as GTR (n=107; 70.9%), STR (n=23; 15.2%), NTR (n=12; 7.9%), and stereotactic or open biopsy (n=9; 6.0%). Median OS was 19.7 months for GTR, 24.1 months for STR, 10.5 months for NTR, and 17.9 months for biopsy. The differences across categories did not reach statistical significance on univariate analysis (log-rank p=0.620), likely reflecting both the modest subgroup sizes and treatment-selection effects, in which surgeons prioritised GTR for circumscribed high-grade tumours and accepted lesser resections for diffuse or eloquent-location tumours [12,13]. The corresponding Kaplan-Meier curves are presented in Figure 5.

Survival by radiotherapy status

**Figure 6:**
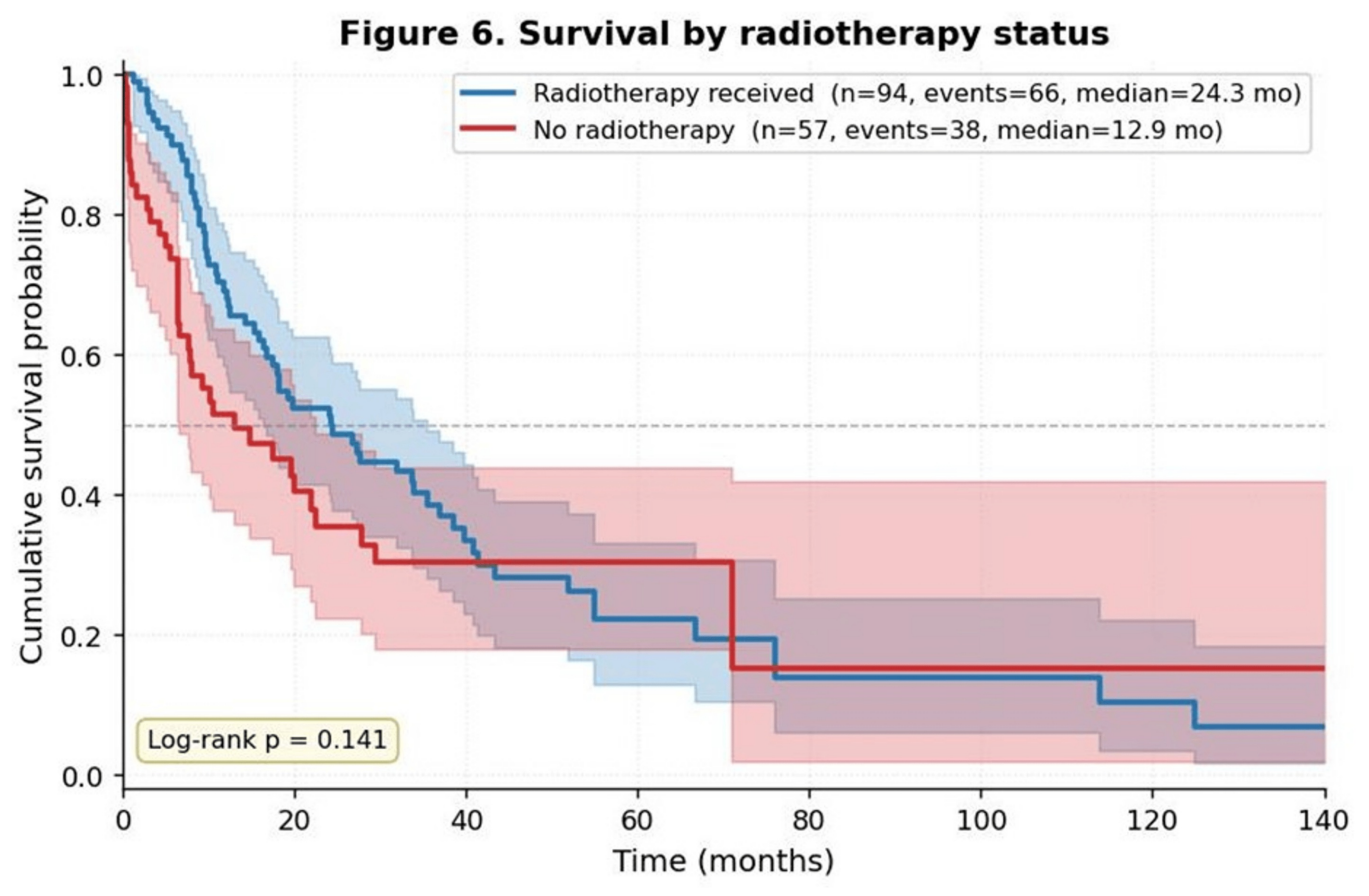
Kaplan-Meier overall survival curves stratified by adjuvant radiotherapy status Strata: radiotherapy received (n=94; 66 events; median: 24.3 months) versus no radiotherapy (n=57; 38 events; median: 12.9 months). Univariate log-rank p=0.141; shaded bands represent 95% confidence intervals.

Of the 151 adult patients, 94 (62.3%) received adjuvant external-beam radiotherapy with prescription doses ranging from 45 to 60 Gy in conventional fractionation, while 57 (37.7%) patients did not receive any radiotherapy. Patients who received radiotherapy had a median OS of 24.3 months compared with 12.9 months for those who did not, an absolute difference of 11.4 months that did not reach statistical significance on univariate analysis (log-rank p=0.141). This non-significant univariate result reflects substantial confounding because patients with the poorest baseline prognosis (older age, deep midline tumours, poor performance status) were also least likely to receive or complete radiotherapy. The numerical estimates are summarised in Table 4, and the corresponding Kaplan-Meier curves are presented in Figure 6.

**Table 4:** Overall survival of adult patients with CNS malignancies by radiotherapy status Univariate log-rank p=0.141. See the Discussion for confounding considerations. CI, confidence interval; CNS, central nervous system; OS, overall survival.

Radiotherapy status	n	Events	Median OS (months)	95% CI
Radiotherapy received	94	66	24.3	16.3-35.5
No radiotherapy	57	38	12.9	6.5-22.4

Survival by adjuvant chemotherapy

Adjuvant systemic therapy, predominantly temozolomide (Cycle 1: oral: 150 mg/m^2^ once daily on days 1 to 5 of the 28-day treatment cycle. Cycles 2 to 6: oral: may increase to 200 mg/m^2^ once daily on days 1 to 5; repeat every 28 days for a total of 6 cycles), was administered to 52 (34.4%) patients; 99 (65.6%) patients received no adjuvant chemotherapy. The median OS was 26.8 months among patients who received chemotherapy compared with 15.2 months for those who did not (log-rank p=0.340). Detailed chemotherapy dosing, dose reductions, and cycle completion data were not consistently retrievable from the retrospective database and thus could not be included in the analysis.

As with radiotherapy, the absence of a univariate significance signal reflects the strong negative selection that operated in this cohort, in which financial barriers, treatment abandonment, and poor performance status disproportionately reduced chemotherapy uptake among patients with the worst baseline prognosis.

Multivariable Cox proportional hazards analysis

A multivariable Cox proportional hazards model was fitted with age (per 10-year increment), male sex, WHO grade IV status, receipt of radiotherapy, receipt of adjuvant chemotherapy, and gross resection (GTR or STR) as covariates. Each additional decade of age conferred a 1.84-fold increase in mortality risk (HR: 1.84, 95% CI: 1.52-2.23, p<0.001), making age the only variable that reached independent statistical significance. Grade IV status showed a non-significant trend (HR: 1.47, 95% CI: 0.92-2.34, p=0.109). Receipt of radiotherapy, chemotherapy, and gross resection all showed point estimates favouring treatment (HR: 0.78, 0.75, and 0.74, respectively) but did not achieve statistical significance, consistent with treatment-selection confounding. The model concordance index was 0.72. The full model is summarised in Table 5.

**Table 5:** Multivariable Cox proportional hazards model for overall survival in adult patients with CNS malignancies Number of patients in the model: 151; number of events: 104. Model concordance index=0.72 CI: confidence interval; CNS, central nervous system; GTR: gross total resection; HR: hazard ratio; STR: subtotal resection; WHO: World Health Organization.

Covariate	Adjusted HR	95% CI	p-value
Age (per 10-year increment)	1.84	1.52-2.23	<0.001
Male sex	1.09	0.72-1.66	0.670
WHO grade IV (vs. grade II-III)	1.47	0.92-2.34	0.109
Adjuvant radiotherapy received	0.78	0.49-1.25	0.308
Adjuvant chemotherapy received	0.75	0.47-1.19	0.226
Gross resection (GTR or STR)	0.74	0.40-1.35	0.324

## Discussion

This single-centre retrospective cohort study provides one of the first dedicated longitudinal survival analyses of adult primary CNS malignancies from a tertiary hospital in Bangladesh. By restricting the cohort to adult patients aged 18 years or older and reporting outcomes against the contemporary WHO 2021 framework, the study delivers a benchmark dataset for a resource-constrained setting where comparable data have until now been scarce.

Our median OS of 19.6 months for the whole adult cohort is consistent with published mixed-histology series from neighbouring South Asia, where tertiary-hospital cohorts have reported aggregate medians in the 18-22-month range [4]. The figure remains substantially below contemporary population-level estimates from HIC registries, in which five-year relative survival for malignant primary CNS tumours has improved with the integration of molecular diagnostics, concurrent chemoradiation, and surgical-navigation technologies [14]. Several factors specific to LMIC settings plausibly explain this gap, including delayed presentation with large tumour burden and impaired performance status, financial barriers to completion of the Stupp regimen, and the limited availability of standardised supportive care for tumour-associated complications such as cerebral oedema and venous thromboembolism.

The most clinically important subgroup-level finding concerns the glioblastoma stratum. Our median OS of 12.4 months in this group is notably shorter than the 14.6 months reported by the pivotal European trial that established radiotherapy plus concurrent and adjuvant temozolomide as the global standard of care [15]. Several factors contribute to this gap. First, patients in our setting frequently present at an advanced stage with extensive disease burden owing to delays in symptom recognition and referral [7]. Second, although external-beam radiotherapy is available at the study institution, the full Stupp regimen with concurrent and adjuvant temozolomide remains financially prohibitive for many families, leading to dose reductions, treatment abandonment, or omission of the systemic component altogether. Third, the management of tumour-related complications, including cerebral oedema, perioperative seizures, and thromboembolic events, is less protocol-driven in our environment, which can contribute to early mortality before any survival benefit from oncological therapy can accrue.

A critical methodological consideration in interpreting the glioblastoma result is the definition of the entity under the 2021 WHO classification. Under that framework, the term "glioblastoma" is now reserved exclusively for IDH-wildtype tumours, whereas IDH-mutant tumours with similar histological features are designated "astrocytoma, IDH-mutant, grade IV" [3]. Because routine molecular testing was not available at our centre, the glioblastoma category in this study probably includes a small fraction of IDH-mutant cases that would today be reclassified into the astrocytoma group with a more favourable prognosis. Paradoxically, the inclusion of better-prognosis IDH-mutant cases should bias our reported glioblastoma survival upwards. The fact that the observed median is nonetheless only 12.4 months suggests that survival for true IDH-wildtype glioblastoma in this setting may be even shorter, reinforcing the case for routine immunohistochemical surrogates (such as the IDH1-R132H antibody) in Bangladeshi neuropathology laboratories [16].

The histopathological distribution observed here, with glioblastoma accounting for slightly more than half of malignant cases, lower-grade astrocytoma for roughly one-third, and oligodendroglioma and ependymoma for the remainder, broadly mirrors patterns reported by the United States Central Brain Tumor Registry [12] and is consistent with previous Bangladeshi hospital-based series [6,7]. The exceptional median OS observed in the oligodendroglioma stratum (153.3 months) aligns with the recognised indolent biology of these tumours, particularly when they harbour 1p/19q codeletion [11], although the absence of routine molecular profiling means that the proportion of true codeleted cases in our subgroup cannot be quantified.

The dominant role of age in our multivariable analysis (HR: 1.84 per 10-year increment, p<0.001) reproduces a long-recognised pattern in adult neuro-oncology. Patients aged 60 years or older in our cohort had a median OS of only 7.7 months, comparable to the four-to-eight-month range reported for elderly patients with glioblastoma even in HICs [15]. By contrast, young adult patients aged 18-40 years achieved a median OS of 66.7 months, reflecting the well-documented advantage conferred by both more favourable molecular tumour biology and greater physiological tolerance of multimodal therapy. The absence of formal performance status data (Karnofsky Performance Status or Eastern Cooperative Oncology Group Performance Status) in our records is a recognised limitation; performance status is an established independent prognostic factor in neuro-oncology, and its retrospective extraction from clinical narrative was not feasible with adequate fidelity for the entire cohort.

Beyond clinical interpretation, our findings carry health-system implications. The pronounced apparent survival benefit associated with adjuvant radiotherapy (24.3 versus 12.9 months on univariate analysis) supports continued and expanded investment in radiotherapy capacity, even though the univariate difference did not reach statistical significance after accounting for treatment-selection effects. Similarly, the directionally favourable adjusted HRs for radiotherapy, chemotherapy, and gross resection are consistent with a treatment effect that this cohort is underpowered to detect with full confidence. Patient and family counselling in this region also benefits from culturally and linguistically attuned communication; for example, simple structured advice to maintain adequate oral hydration with water (pani) and to monitor for steroid-related complications is more readily adopted when delivered in locally familiar terms.

Strengths and limitations

Key strengths include the strictly adult cohort definition, the use of WHO 2021 terminology, explicit framing of comorbidity exclusions using condition-specific staging systems, reporting of follow-up completeness and censoring rate, and adherence to STROBE reporting conventions. Several limitations warrant emphasis. First, as a single-centre series from a tertiary referral hospital, the cohort is subject to referral and financial selection bias. Second, the absence of routine molecular diagnostics (IDH, 1p/19q, MGMT) prevents WHO 2021-compliant integrated diagnosis; all subtype classifications should be interpreted as morphological approximations. Third, the retrospective design constrains data completeness, with performance status, steroid use, antiepileptic prophylaxis, and salvage therapy not consistently retrievable; the absence of performance status data is particularly consequential, as it is one of the strongest determinants of both treatment eligibility and survival in neuro-oncology, and its omission almost certainly biases the treatment-related HRs. Fourth, the modest size of several histopathological subgroups (ependymoma, oligodendroglioma) yields wide CIs for the corresponding survival estimates, and the reported medians for these groups are statistically fragile. Fifth, detailed chemotherapy dosing, dose reductions, and cycle completion data were not consistently retrievable. Sixth, the multivariable model is limited by the moderate number of events relative to candidate covariates, and unmeasured confounders such as performance status and socioeconomic status may bias the treatment-related HRs.

Future directions

Three priorities emerge for neuro-oncology research and service development in Bangladesh. First, the establishment of a national cancer registry that integrates major public and private centres is essential to capture true epidemiological burden and to move beyond single-centre observational work [5]. Second, cost-effective immunohistochemical surrogates for molecular markers, in particular IDH1-R132H, ATRX, and p53, should become standard in all tertiary neuropathology laboratories [16]. Third, prospective multicentre studies should evaluate hypofractionated radiotherapy schedules (for example, 40 Gy in 15 fractions) and abbreviated temozolomide regimens for elderly and poor-prognosis patients with glioblastoma, with a view to reducing treatment-abandonment rates in financially constrained households [17].

## Conclusions

This single-centre adult cohort study from a tertiary hospital in Bangladesh demonstrates that OS from primary CNS malignancies remains substantially lower than benchmarks from high-income settings, with a median OS of 19.6 months across the cohort and 12.4 months for glioblastoma. Age was the dominant independent prognostic factor on multivariable analysis, with each additional decade of life conferring an 84% increase in mortality risk. Treatment-related variables, including radiotherapy, adjuvant chemotherapy, and gross surgical resection, showed directionally favourable effects that did not reach independent significance, consistent with treatment-selection confounding rather than therapeutic ineffectiveness. The findings reinforce the urgent need for routine molecular diagnostics, expanded radiotherapy and chemotherapy capacity, formal capture of performance status in clinical records, and a national cancer registry to enable evidence-based neuro-oncological care in Bangladesh.

## Appendices

Appendix A

**Table 6:** Schoenfeld residual test: proportional hazards assumption GTR: gross total resection; STR: subtotal resection; WHO: World Health Organization.

Covariate	Chi-squared (χ²)	df	p-value	PH assumption
Age (per 10-year increment)	1.58	1	0.209	Satisfied ✓
Male sex	0.94	1	0.332	Satisfied ✓
WHO grade IV (vs. grade II-III)	0.91	1	0.341	Satisfied ✓
Adjuvant radiotherapy received	1.78	1	0.182	Satisfied ✓
Adjuvant chemotherapy received	1.23	1	0.267	Satisfied ✓
Gross resection (GTR or STR)	0.62	1	0.432	Satisfied ✓
Global test	6.84	6	0.335	Satisfied ✓
